# Effects of Hypotonic and Isotonic Enteral Electrolyte Solutions Administered in Continuous Flow in Weaned Foals

**DOI:** 10.3389/fvets.2020.00280

**Published:** 2020-05-22

**Authors:** Lorena Chaves Monteiro, Rinaldo Batista Viana, Marcel Ferreira Bastos Avanza, Pedro Ancelmo Nunes Ermita, Caio Monteiro Costa, Samuel Rodrigues Alves, Paulo Vinícius de Morais Santos, Micheline Ozana da Silva, Daniel Atila de Barros Balbino, Felipe Sperandio de Mattos, Raffaella Bertoni Cavalcanti Teixeira, José Dantas Ribeiro Filho

**Affiliations:** ^1^Laborary of Research in Veterinary Internal Medicine, Veterinary Department, Universidade Federal de Viçosa, Viçosa, Brazil; ^2^Institute of Animal Health and Production, Universidade Federal Rural da Amazônia, Belém, Brazil; ^3^Instituto de Estudo dos Trópicos Úmidos, Universidade Federal do Sul e Sudeste do Pará, Xinguara, Brazil; ^4^Veterinary Clinic and Surgery Department, Universidade Federal de Minas Gerais, Belo Horizonte, Brazil

**Keywords:** maintenance solutions, low sodium, osmolarity, electrolyte replacement, fluid therapy

## Abstract

The effects of fluid therapy with maintenance enteral electrolytic solutions administered by nasogastric route in continuous flow have not previously been studied in weaned foals. This study primary goal was to compare the effects of two maintenance enteral electrolytic solutions administered by nasogastric route in continuous flow on the hydro electrolytic balance in weaned foals. This paper was a controlled trial in a cross-over design (6 × 2) performed in six foals with a mean age of 7.3 ± 1.4 months; each animal received two treatments, IsoES and HypoES, with an interval of 7 days between treatments. After 12 h of fasting, the animals were treated with enteral electrolyte solutions administered via nasogastric route in continuous flow in a volume of 15 mL/kg/h for 12 h. The evaluations were performed at T-12h (the beginning of the fasting), T0h (end of fasting and beginning of fluid therapy), T4h (4 h of fluid therapy), T8h (8 h of fluid therapy), T12h (end of fluid therapy), and T24h (12 h after the end of fluid therapy). Twelve hours of fasting resulted in a reduction (*P* < 0.05) in body weight, abdominal circumference, whereas serum sodium, SID and enophthalmos increased. Twelve hours of fluid therapy normalized these parameters and promoted increased urinary volume and decreased urinary density without causing electrolyte imbalances. Both enteral electrolytic solutions were effective in reestablishing clinical and laboratorial variables without causing electrolyte imbalances.

## Introduction

Foals are more sensitive to hydro electrolytic imbalances than adult horses because they have a higher percent of water in the body, higher metabolic rates, and greater insensible water losses (1). The first year of the foal's life is the most critical period, with the greatest morbidity. Diarrhea, enteritis and bronchopneumonia are the main diseases that affect these animals and trigger hydro electrolytic and base-acid imbalances, and dehydration (2, 3). The presence of these changes makes it mandatory to use fluid therapy with electrolytic solutions to correct these disorders.

In horses, enteral fluid therapy (EFT) by nasogastric route in bolus or continuous flow is an important hydration method. It is effective for patient rehydration, expansion of blood volume, and correction of electrolyte and acid-base imbalances in various animal species (4–7) and humans (8, 9). Based on the authors' clinical experience, EFT is a safe and effective method to correct dehydration in foals with adequate functioning of the gastrointestinal tract. However, it is not commonly used in foals, mainly in neonates, because reports of its use are not widespread in the literature and mainly because there are not commercial products available, due to an absence of experimental studies on maintenance enteral electrolyte solutions in foals.

Historically, it was assumed that isotonic electrolyte solutions with plasma-like osmolarity would improve intestinal absorption. Recent studies have shown that decreased tonicity of enteral rehydration solutions resulted in better effects on water and electrolyte absorption without causing adverse effects (10, 11).

There are no experimental studies in the literature evaluating isotonic or hypotonic enteral electrolyte solutions in foals and for this reason their maintenance fluid therapy requirements are unknown. On the other hand, studies in adult horses and animals of other species indicate that enteral maintenance electrolyte solutions should contain a lower concentration of sodium than plasma. Since foals can easily develop hypernatremia when receiving isotonic solutions, a study evaluating an electrolyte solution containing less sodium may open a new perspective for fluid therapy in foals. The aims of the study reported here were to compare the effects of two enteral electrolyte solutions containing different osmolarities on the hydro electrolytic balance of weaned foals.

## Materials and Methods

### Experimental Design

This paper was a controlled trial in a cross-over design. Six healthy foals of the Mangalarga Marchador breed, of both sexes, with mean age of 7.3 ± 1.4 months and mean body weight of 165 ± 24.4 kg, were used in this study. All animals were considered healthy based on clinical and laboratory tests. The foals were kept in a paddock, fed concentrate (1% body weight), and supplied with Tyfton 85 hay, water, and mineral supplementation ad libitum.

The effects of two treatment solutions with the following compositions were evaluated: Isotonic Enteral Electrolyte Solution (IsoES): 4 g/L sodium chloride; 0.5 g/L potassium chloride, 0.3 g/L magnesium chloride hexahydrate, 2 g/L calcium acetate monohydrate, 4 g/L sodium acetate trihydrate and 10-g/L dextrose, with an osmolarity of 289 mOsm/L; and Hypotonic Enteral Electrolyte Solution (HypoES): 4 g/L sodium chloride; 0.5 g/L potassium chloride, 0.3 g/L magnesium chloride hexahydrate, 2 g/L calcium acetate monohydrate and 10 g/L dextrose, with an osmolarity of 225 mOsm/L. The concentrations of each electrolyte (mmol/L) in both treatments are demonstrated in Table 1.

**Table 1:** Components of enteral isotonic (IsoES) and hypotonic (HypoES) electrolyte solutions administered in continuous flow in foals.

**Treatments**	**IsoES**	**HypoES**
Sodium (mmol L^−1^)	107	73
Potassium (mmol L^−1^)	6.5	6.5
Chloride (mmol L^−1^)	89.3	89.3
Calcium (mmol L^−1^)	4.34	4.34
Magnesium (mmol L^−1^)	1.16	1.16
Acetate (mmol L^−1^)	52	22.6
Glucose (mmol L^−1^)	55.5	55.5
Measured Osmolarity (mOsm L^−1^)	289	225
SID (mmol L^−1^)	24.2	−9.8

**Figure 1:**
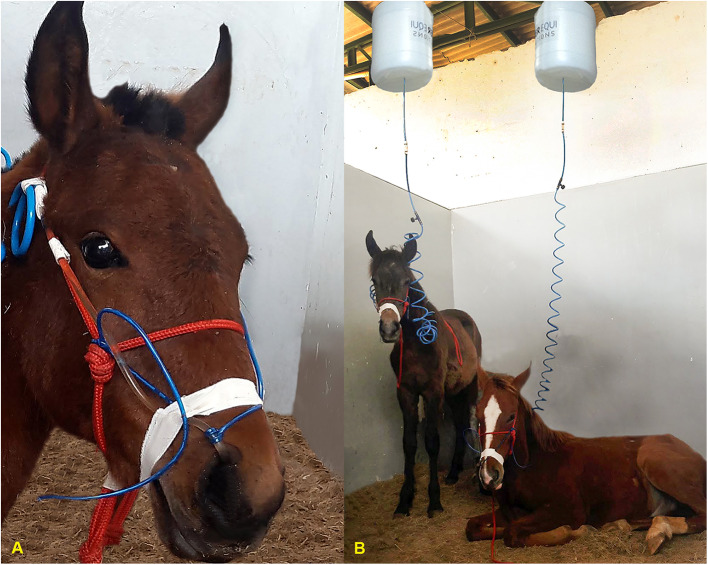
**(A)** Highlight for the fixation technique of the nasogastric probe to the halter and face of the animals. **(B)** Animals during enteral fluid therapy phase.

The animals were randomly assigned into treatment groups in a cross-over design (6x2). Each animal received both treatments with intervals of seven days between them; this ensured that there was no overlap of effects. During the experimental period the animals were kept in stalls with dimensions of 4 x 4 meters. Before the start of fluid therapy, the animals were fasted from food and water for 12 h and kept in stalls with rubber mats to avoid bed intake. After the fasting period, a nasogastric tube was inserted (5 mm of internal diameter x 6 mm of external diameter and 1.5 m in length). The presence of the nasogastric tube in the stomach was confirmed by the return of gastric contents after aspiration. The tube was attached to the halter and connected to the enteral fluid therapy system, consisting of a reservoir with a 20 liters capacity connected to a 5-meter-long polyurethane coil infusion set with a drip chamber and a flow regulator (Figure 1).

Both treatments were administered for 12 h in a continuous flow at 15 mL/kg/h, with the animals kept in stalls. The rate of 15 mL/kg/h was based on our clinical routine, in human medicine (12) and veterinary medicine clinical trials (5, 12–14). After the end of the fluid therapy period, the foals were released in a paddock where they received concentrate (at 0.5% body weight), Tifton 85 hay, water and mineral supplementation ad libitum.

### Clinical Evaluations and Collection of Biological Samples

Clinical and laboratory evaluations were performed at the beginning of the fasting phase (T-12h), at starting of the fluid therapy phase (T0h), at 4 h (T4h), at 8 h (T8h) after fluid therapy began, at the end of fluid therapy (T12h), and 12 h after the end of fluid therapy (T24h). The clinical evaluation of the animals consisted of a determination of body weight (BW) measured on a mechanical scale, abdominal circumference (AC) measured with a tape measure in the paralumbar fossae at the 17th intercostal space, and the degree of enophthalmos (DE) classified into the following scores, as was described by Tremblay (1990): enophthalmos absent when there was no gap between the eyeball and the eye orbit (0), mild enophthalmos when there was a small noticeable gap between the eyeball and the eye orbit (1), moderate enophthalmos when there was a substantial gap between the eyeball and the eye orbit (2), intense enophthalmos when the eyes were deeply sunk in the eye orbit (3).

Blood samples were collected via jugular venipuncture with a vacuum system in tubes containing EDTA K2 for globular volume determination, performed by microhematocrit technique. To obtain serum, blood samples were collected in tubes with clot activator and were kept in a water bath at 37°C for 40 minutes for clot formation. Serum separation was performed by centrifugation and then stored at −20°C until analysis. Serum osmolarity was determined by freezing point depression (Osmometer 3320, Advanced Instruments Inc, Massachusetts, USA). Serum sodium (Na^+^) and potassium (K^+^) concentrations were determined by flame photometry technique (Photometer B462, Micronal, São Paulo, Brazil). Measurement of serum chloride (Cl^−^) concentrations (Mercury Thiocyanate, Bioclin Quibasa, Minas Gerais, Brazil), total calcium (tCa^2+^) concentrations (Arsenazo III, Bioclin Quibasa, Minas Gerais, Brazil) and total magnesium (tMg^2+^) concentrations (Xylidyl Blue, Bioclin Quibasa, Minas Gerais, Brazil) were performed in an automatic clinical chemistry device (HumaStar 300 Automated Chemistry Analyzer, Human Diagnostics, Wiesbaden, Germany).

The collection of urine and feces were performed by spontaneous urination and defecation. The authors remained with the animals throughout the fluid therapy phase to collect these materials. In each urination, all the volume of urine produced was collected in buckets previously cleaned with distilled water and dried. All feces produced were collected in previously sanitized and dried plastic trays. At each urine and feces collection point the time and the total volume produced were recorded, then a sample was taken for analysis.

Urine and feces produced were collected at the moments: T-12h (urine obtained from one urination immediately before the onset of fasting from water and food); T0-2h (all the urine and feces produced in the first 2 h of fluid therapy); T2-6h (all urine and feces produced between 2 and 6 h of fluid therapy); T6-10h (all urine and feces produced between 6 and 10 h of fluid therapy); T10-12h (all urine and feces produced between 10 and 12 h of fluid therapy); and T24h (urine obtained from a urination 12 h after the end of fluid therapy).

The urinary volume produced in each of these intervals and the volume of water eliminated in the feces were divided by the hours of each interval (T0-2h/2 hours; T2-6h/4 hours; T6-10h/4 hours; T10-12h/2 hours). From this, the value of urinary output (mL/hour) and water loss in the feces of each animal, at each time and in each treatment was obtained. Urine analysis determined all volume of urine produced in each moment of urination, urine specific gravity (USG), and urinary excretion of sodium and potassium (Photometer B462, Micronal, São Paulo, Brazil), chloride, total calcium, and total magnesium.

### Calculations

The determination of the strong ion difference (SID) values was performed using the serum concentrations of strong ions and according to the following equation (15):

$$\begin{array}{l} SID\;\left(mmol/L\right)=\left[\left(Na^{+}\right)+\left(K^{+}\right)\right]-\;Cl^{-} \end{array}$$

The urinary excretion of electrolytes (C) was calculated according to the following formula:

$$\begin{array}{l} C=U_{x}*V\; \end{array}$$

where Ux is the mean concentration of the substance in the urine (mg/mL or mmol/mL) and V is the mean urinary flow at each time (mL/minute).

The volume of water in the feces was calculated by multiplying the total weight of feces produced in each defecation by its moisture content. To determine the feces moisture content (FMC) the samples were weighed in an aluminum tray (F1) and kept in a kiln at 80 °C until weight stabilization (F2). Then the feces moisture content was calculated by the difference between F1 and F2 according to the formula:

$$\begin{array}{l} FMC=\;\frac{F_{1}-F_{2}}{F_{1}}*100 \end{array}$$

At each defecation the total volume of water in feces (WF) was determined by multiplying the FMC by the total weight of the feces (TWF).

$$\begin{array}{l} WF=FMC*TWF \end{array}$$

### Statistical Analysis

Data were subjected to descriptive analysis to obtain means and standard deviations. The normality of the data distribution and the sphericity of the variances were evaluated with Shapiro-Wilk and Mauchly tests, respectively. The main effects of time, treatments, and interaction time ^*^ treatment were evaluated with an ANOVA based on a factorial planning of repeated measures. When necessary, a *post hoc* test of Least Significant Difference (LSD) was used to determine significance. For the variables that did not meet the ANOVA assumptions, the time effect was evaluated with a Kruskal-Wallis non-parametric test followed by Dunn's *post hoc* test, and the effect of the treatment at each time was evaluated with a Wilcoxon's test. All analyses were performed with the SPSS 20 (IBM, SPSS, Chicago, USA) statistical package, and P values <0.05 were considered significant.

## Results

The nasogastric tube was well tolerated by the animals, the technique used to attach the tube to the halter was effective and no repetition of the procedure was necessary during the experimental phase.

After 12 h of fasting, at the beginning of the fluid therapy phase (T0h), the animals of both groups showed a decrease in body weight (Table 2). The body weight returned to values similar to T-12h after 4 h of fluid therapy (T4h) and remained stable until the end of the experiment (T24h). Both groups presented with reduction of the abdominal circumference at T0h (Table 2). At T4h the abdominal circumference returned to the values observed at T-12h, remaining stable until T24h. All animals developed mild to moderate enophthalmos (*P* < 0.05) after fasting (Table 2). This parameter normalized 4 h (T4h) after the beginning of the fluid therapy period and remained constant throughout the observed period.

**Table 2:** Mean values and standard deviations of the clinical parameters [body weight (BW), abdominal circumference (AC), packed cell volume (PCV) and total serum protein (TP)] and median with interquartile range (0.25–0.75) of the degree of enophthalmos (DE) of foals hydrated with isotonic and hypotonic electrolyte solutions delivered in continuous flow.

**Variable**	**Groups**	**Moments**					
		**Fasting**	**Fluid therapy**				**Clinical observation**
		**T-12h**	**T0h**	**T4h**	**T8h**	**T12h**	**T24h**
BW (kg)	IsoES	166 ± 25^a^	158 ± 23^b^	163 ± 22^ab^	164 ± 23^a^	166 ± 22^a^	164 ± 22^ab^
	HypoES	166 ± 22^a^	158 ± 23^b^	163 ± 22^a^	162 ±22^ab^	162 ± 22^ab^	163 ± 23^ab^
AC (cm)	IsoES	139 ± 7^a^	130 ± 7^b^	135 ± 5^ab^	139 ± 7^ab^	138 ± 7^a^	136 ± 10^ab^
	HypoES	139 ± 6ª	131 ± 6^b^	134 ± 6^ab^	136 ± 7^ab^	137 ± 6^ab^	134 ± 5^ab^
PCV (%)	IsoES	29 ± 1.1	31.2 ± 2.5	31.3 ± 4.7	31.2 ± 3.5	30.2 ± 4.0	31 ± 4.8
	HypoES	29.2 ± 2.1	31.2 ± 4	30.8 ± 2.9	32.2 ± 3.8	31 ± 3.6	31.2 ± 3.5
TP (g/dL)	IsoES	5.7 ± 0.4	6.0 ± 0.4	5.8 ± 0.5	5.9 ± 0.3	5.9 ± 0.4	5.9 ± 0.3
	HypoES	5.8 ± 0.4	6.1 ± 0.3	5.9 ± 0.6	6.0 ± 0.6	6.0 ± 0.3	5.7 ± 0.6
DE^*^	IsoES	0 (0)^b^	1.5 (1.25)^a^	0 (0.5)^ab^	0 (0.5)^ab^	0 (0)^b^	0 (0.25)^ab^
	HypoES	0 (0)^b^	1.5 (1.0)^a^	0 (0.25)^b^	0 (1.0)^ab^	0 (0.25)^b^	0 (1.25)^ab^

ANOVA based on a factorial planning of repeated measures. Means followed by different superscripted lower-case letters on the same line differ between time-points by LSD test (P < 0.05).

* Kruskal–Wallis non-parametric test. Median followed by different superscripted lower-case letters on the same line differ between time-points by Dunn's test (P < 0.05).

*There was no significant difference between groups for these variables*.

Serum sodium increased (*P* < 0.05) at T0h in both groups and remained high until T12h of fluid therapy (T12h) in the IsoES group. In the HypoES group, however, there was a small reduction (*P* < 0.05) at T12h (Table 3). There was no difference (*P* > 0.05) in the osmolarity results (Table 3).

**Table 3:** Mean values and standard deviations of the serum biochemical profile [sodium (Na^+^), serum osmolarity (OSM), potassium (K^+^), total calcium (tCa^2+^), chloride (Cl^−^), total magnesium (tMg^2+^), and strong ion difference (SID)] of foals hydrated with isotonic and hypotonic electrolyte solutions delivered in continuous flow.

**Variable**	**Groups**	**Moments**					
		**Fasting**	**Fluid therapy**				**Clinical observation**
		**T-12h**	**T0h**	**T4h**	**T8h**	**T12h**	**T24h**
Na^+^ (mmol/L)	IsoES	131 ± 3.9^bd^	137 ± 4.6^ac^	135 ± 2.8^ab^	134 ± 3.6^abc^	134 ± 3.8^abc^	132 ± 3.4^cd^
	HypoES	135 ± 2.8^ab^	136 ± 2.3^a^	135 ± 3.5^a^	133 ± 4.6^ab^	130 ± 3.4^b^	134 ± 2.5^a^
Osm (mOsm/L)	IsoES	285 ± 2.0	288 ± 4.1	289 ± 0.6	290 ± 8.0	286 ± 2.9	284 ± 5.4
	HypoES	281 ± 1.0	287 ± 3.9	288 ± 5.5	285 ± 6.4	280 ± 2.8	284 ± 3.1
K^+^ (mmol/L)	IsoES	4.8 ± 0.4^a^	4.0 ± 0.5^bc^	3.9 ± 0.5^b^	3.7 ± 0.5^bc^	3.5 ± 0.5^bc^	4.3 ± 0.5^ac^
	HypoES	4.7 ± 0.2^a^	4.0 ± 0.3^b^	4.3 ± 1.1^ab^	3.9 ± 0.5^bc^	3.8 ± 0.4^b^	4.7 ± 0.5^ac^
tCa^2+^ (mmol/L)	IsoES	2.38 ± 0.08	2.33 ± 0.2	2.65 ± 0.35	2.45 ± 0.08	2.63 ± 0.25	2.45 ± 0.2
	HypoES	2.33 ± 0.13	2.23 ± 0.18	2.2 ± 0.25	2.48 ± 0.13	2.4 ± 0.1	2.45 ± 0.15
Cl^−^ (mmol/L)	IsoES	92.9 ± 2.7	93.5 ± 1.0	94.7 ± 1.1	95.4 ± 2.0	95.9 ± 2.2	93.6 ± 3.3
	HypoES	94.8 ± 2.5	92.0 ± 2.0	90.6 ± 2.6	95.0 ± 1.7	92.2 ± 2.4	92.9 ± 3.1
tMg^2+^ (mmol/L)	IsoES	0.91 ± 0.07^a^	0.79 ± 0.03^b^	0.7 ± 0.04^ce^	0.58 ± 0.09^d^	0.58 ± 0.13^cd^	0.77 ± 0.11^be^
	HypoES	0.88 ± 0.12^a^	0.74 ± 0.07^b^	0.65 ± 0.08^c^	0.61 ± 0.05^c^	0.60 ± 0.06^c^	0.88 ± 0.09^a^
SID (mmol/L)	IsoES	43.2 ± 2.5^ab^	48.1 ± 4.6^a^	45.0 ± 2.2^ab^	41.9 ± 2.7^b^	44.0 ± 6.2^ab^	44.0 ± 2.7^ab^
	HypoES	46.4 ± 3.6^ab^	48.2 ± 3.0^a^	46.0 ± 4.3^ab^	42.2 ± 4.4^b^	41.7 ± 4.4^b^	42.5 ± 8.8^b^

*ANOVA based on a factorial planning of repeated measures. Means followed by different superscripted lower-case letters on the same line differ between time-points by LSD test (P < 0.05). There was no significant difference between groups for these variables*.

Both groups showed reduction (*P* < 0.05) of serum potassium concentrations at T0h, this decrease remained until T12h (Table 3). However, at T8h the animals of the HypoES group showed a slight increase (*P* < 0.05) in this blood electrolyte. At T24h the serum potassium concentrations in both treatment groups returned to the initial values noted at T-12h (*P* < 0.05). There was no difference (P > 0.05) in the serum total calcium and chloride concentrations (Table 3). There was a progressive reduction (*P* < 0.05) in serum total magnesium concentrations from T0h, but there was no difference (*P* > 0.05) in this decrease between treatments (Table 3). At T24h, in both groups there was an increase (*P* < 0.05) in serum total magnesium concentration, but in the HypoES the values returned (*P* < 0.05) to similar results at T-12h (Table 3). The SID did not differ between treatments, but significant differences (*P* < 0.05) were identified in both treatments over time. An increase was observed in T0h and a decrease in T8h.

Urine specific gravity differed (*P* < 0.05) between treatments and times (Table 4). In the T2-6h period, the urine specific gravity values of both groups decreased (*P* < 0.05). The HypoES maintained the lowest values during the whole fluid therapy period, and the resultant values were significantly different (*P* < 0.05) from those observed with IsoES in the T2-6h and T10-12h periods.

**Table 4:** Mean values and standard deviations of the urine specific gravity (USG) of foals hydrated with isotonic and hypotonic electrolyte solutions delivered in continuous flow.

**Variable**	**Groups**	**Moments**					
		**Fasting**	**Fluid therapy**				**Clinical observation**
		**T-12h**	**T0-2h**	**T2-6h**	**T6-10h**	**T10-12h**	**T24h**
USG	IsoES	1,037 ± 1^Aad^	1,042 ± 8^Aa^	1012 ± 8^Acd^	1,005 ± 4^Ab^	1,008 ± 3^Abc^	1,024 ± 4^Aad^
	HypoES	1,036 ± 6^Aa^	1,033 ± 17^Aa^	1,007 ± 8^Bbc^	1,005 ± 3^Abd^	1,004 ± 2^Bd^	1,025 ± 4^Aac^

*ANOVA based on a factorial planning of repeated measures. Means followed by different superscripted lower-case letters on the same line differ between time-points by LSD test (P < 0.05). Means followed by different superscripted upper-case letters in the same column indicate significant differences between treatments by LSD test (P < 0.05)*.

The volume of water in feces did not show significant difference between treatments and over time (*P* > 0.05). However, at T12h the water in feces in the IsoES treatment was almost twice higher as in the HypoES group (Table 5). Urinary volume increased (*P* < 0.05) in the groups during the fluid therapy period (T0h to T12h), but there was no difference between treatments (Table 5).

**Table 5:** Mean values and standard deviations of the of the volume of water in the feces (WF) and urinary biochemical profile [urinary volume (UV), urinary excretion of sodium during the hydration period (urNa^+^), potassium (urK^+^), total calcium (urCa^2+^), chloride (urCl^−^) and total magnesium (urMg^2+^)] of foals hydrated with isotonic and hypotonic electrolyte solutions delivered in continuous flow.

**Variable**	**Groups**	**Moments**			
		**Fluid therapy**			
		**T0–2h**	**T2–6h**	**T6–10h**	**T10–12h**
WF (mL/hour)	IsoES	358 ± 96	375 ± 30	364 ± 71	900 ± 694
	HypoES	510 ± 368	491 ± 162	320 ± 140	510 ± 243
UV (mL/hour)	IsoES	211 ± 38^b^	676 ± 566^ab^	1289 ± 606^a^	858 ± 224^a^
	HypoES	257 ± 45^b^	1040 ± 233^a^	1590 ± 384^a^	1003 ± 227^a^
urNa^+^ (mmol/min)	IsoES	0.40 ± 0.19^b^	0.95 ± 0.68^ab^	1.65 ± 1.01^a^	1.20 ± 0.45^a^
	HypoES	0.53 ± 0.27^b^	1.13 ± 0.30^b^	1.64 ± 0.47^a^	1.00 ± 0.30^b^
urK^+^ (mmol/min)	IsoES	0.64 ± 0.17^a^	0.40 ± 0.30^ab^	0.42 ± 0.24^ab^	0.25 ± 0.11^b^
	HypoES	0.80 ± 0.34^a^	0.51 ± 0.35^bc^	0.56 ± 0.17^ac^	0.18 ± 0.06^b^
urCa^2+^ (mg/min)	IsoES	0.92 ± 0.59	3.10 ± 1.94	5.49 ± 1.88	3.78 ± 2.06
	HypoES	1.05 ± 0.36	5.71 ± 2.68	4.18 ± 1.76	3.77 ± 0.90
urCl^−^ (mmol/min)	IsoES	0.80 ± 0.35^b^	1.51 ± 1.19^b^	2.49 ± 1.60^a^	2.12 ± 0.83^ab^
	HypoES	0.70 ± 0.49^c^	2.36 ± 1.0^ab^	2.79 ± 0.80^a^	1.84 ± 0.52^b^
urMg^2+^ (mg/min)	IsoES	1.61 ± 0.50^a^	2.16 ± 1.11^ab^	1.58 ± 0.84^ab^	0.87 ± 0.33^b^
	HypoES	1.33 ± 0.47^b^	2.06 ± 0.50^a^	1.16 ± 0.27^b^	0.67 ± 0.34^c^

*ANOVA based on a factorial planning of repeated measures. Means followed by different superscripted lower-case letters on the same line differ between time-points by LSD test (P < 0.05). There was no significant difference between groups for these variables*.

Urinary sodium excretion did not differ (*P* > 0.05) between treatments (Table 5), but increased in both groups in the T6-10h period (*P* < 0.05). For T10-12h, this increase was maintained in the IsoES group, whereas with HypoES, it returned to the values observed in the T0-2h period (*P* < 0.05). There was no difference (*P* > 0,05) in the urinary excretion of potassium between treatments (Table 5), but in the IsoES group there was a reduction in urinary potassium at T10-12h period, whereas in the HypoES group a reduction was observed in the T2-6h and T10-12h periods (*P* < 0.05).

The urinary excretion of chloride did not differ (*P* > 0.05) between groups (Table 5), but in the IsoES there was an increase in the urinary excretion of chloride during the T6-10h period, which remained until T10-12h. In the HypoES group, this increase occurred at T2-6h period and was maintained until T6-10h, followed by a small decrease at T10-12h. Urinary excretion of total magnesium did not differ (*P* > 0.05) between groups (Table 5), but decreased (*P* < 0.05) throughout the fluid therapy phase in both treatments, with minimal excretion at T10-12h.

## Discussion

Based on the authors' experience, the nasogastric tube can remain for a few days. Naturally the animals may develop minimal irritation of the mucosa of the nasal cavity, pharynx, larynx or esophagus, but without the development of lesions with clinical significance. Usually when patients are treated with enteral fluid therapy, the tube remains for 12 to 72 h and it is not observed the occurrence of complications due to its presence. In foals, small-caliber nasogastric tubes with 6 mm of external diameter are used, and it allows animals to feed normally during enteral fluid therapy. Therefore, the patients without food restriction are provided with water and food during continuous flow enteral fluid therapy. In the case of lactating foals, they may be kept with the dam to nurse.

At T0h in both groups, there was a reduction in body weight and abdominal circumference whereas the degree of enophthalmos increased (Table 2). These changes were induced by the 12 h of water and food restriction imposed to the animals. Based on body weight, in both groups, the water and food restriction period promoted an average dehydration of 4.8%, classified as mild, without considering the production of fecal matter during this period. Classically it is widely accepted that in cases of dehydration of <5% of body weight, the clinical signs are imperceptible to physical examination. However, the results of the present study showed that the degree of enophthalmos increased significantly at T0h (Table 2), although the degree of dehydration was <5%. This demonstrated that the degree of enophthalmos is very sensitive to changes in hydration status in foals, as has also been seen in new born calves (16).

After 4 h (T4h) of fluid therapy there was a return to baseline BW, an increase in AC and correction of enophthalmos in animals of both groups. The results demonstrate the efficacy of enteral fluid therapy in correcting, in a short time, the dehydration caused by the 12 h of water and food restriction.

The increase observed in the serum sodium concentrations of both groups at T0h was attributed to hemoconcentration caused by the 12 h of fasting. During the fluid therapy phase (T0h to T12h), the serum sodium concentrations was unchanged in the IsoES animals, whereas the HypoES animals showed a mild decrease at T12h (Table 2). Despite this decrease, hyponatremia was not found in the animals, as values remained within the normal range (17). The values of serum osmolarity corroborated this fact, as they remained unchanged (*P* > 0.05) in the animals of the two treatments during the whole experimental phase. This is expected because serum sodium associated with serum chloride represents 90% of serum osmolarity (18). The main electrolyte imbalance observed in foals, especially in neonates, is the development of plasma hypernatremia during intravenous fluid therapy (19). The literature indicate that electrolyte maintenance solutions should contain sodium amounts below plasma concentration, preventing development of hypernatremia in patients (20, 21). However, maintenance fluid therapy requirements in foals are unknown, thus the evaluation of two enteral electrolyte solutions containing different amounts of sodium is a relevant aspect when considering the enteral fluid therapy for sick foals.

In both groups there was an increase in the urinary excretion of sodium from T6-10h which remained throughout the fluid therapy period. This may have occurred due to increase in plasma volume caused by fluid therapy, which inhibits the renin-angiotensin-aldosterone system and triggers the release of natriuretic atrial peptide resulting in increased urinary excretion of sodium (22). As previously mentioned, despite the increased urinary sodium, the animals did not develop serum hyponatremia. Similar results were observed in adult horses, demonstrating that these solutions can be used in patients with mild hyponatremia without aggravating the deficit of this electrolyte (12).

The decrease in serum potassium concentration in both groups at T0h may have been due to the food and water restriction, and dehydration that causes activation of the renin-angiotensin-aldosterone system, releasing the antidiuretic hormone, which promotes the renal resorption of water and sodium, and increases urinary potassium excretion (22). In addition, during the fluid therapy phase (T0h–T12h), the presence of dextrose in the electrolytic solutions may have induced the entry of serum potassium into the cells, decreasing its concentration in blood (23). Despite this, the serum potassium concentration remained in the reference range for the species (17). In adult horses, similar results to the present study have been observed (24). From these results, it was observed that in the enteral fluid therapy of animals with hypokalemia, the electrolyte solutions should have more than 0.5 g/L KCl, and the animals should be constantly monitored.

The decrease in urinary potassium excretion in both groups may be associated with plasma volume expansion caused by fluid therapy, inhibiting the release of aldosterone and reducing renal excretion of potassium (22). In addition, the decrease in serum potassium concentration during the fluid therapy period (T0h–T12h) contributed to the observed results.

Although the solutions had a lower amount of chloride (89 mmol/L) than plasma (90–106 mmol/L) (10, 24), the serum chloride levels of the animals did not change in relation to T-12h. The increase of urinary excretion of chloride can be attributed to the renal mechanisms responsible for its reabsorption. Renal reabsorption of this electrolyte is dependent of the electrochemical gradient generated by the presence of sodium reabsorbed into the peritubular space (25). In the present work, the urinary excretion of sodium and chloride varied similarly in both treatments over time. Also, despite increased urinary excretion, the animals did not develop serum hypochloremia and hyponatremia. These results demonstrate that both solutions will not accentuate electrolytic imbalances when used in animals with low intensity chloride disorders.

Serum total calcium concentration was expected to be elevated, as both treatments contained 4.3 mmol/L, an amount greater than the total serum calcium for foals (1.6–2.5 mmol/L) (17). However, there was no variation between groups (*P* > 0.05) in the serum concentrations of this electrolyte. The same result was observed in urinary excretion of total calcium, although there was an increase in excretion during fluid therapy, this was not significant (*P* > 0.05). The maintenance of the serum ionic calcium concentration was observed in adult horses submitted to enteral fluid therapy with electrolytic solutions (24). The results of the present study demonstrate that the amount of calcium present in the solutions was sufficient to avoid its decrease by hemodilution during fluid therapy without triggering hypercalcemia.

Magnesium homeostasis is mainly regulated by intestinal absorption. Thus, the decrease in the levels of this electrolyte observed at T0 may be related to the fasting (26). Although the two electrolytic solutions contained 1.16 mmol/L magnesium, there was a reduction in the serum total magnesium concentrations during the fluid therapy phase (T0h-T12). Similar results were observed in other studies with adult horses (4, 24, 27) and were related to the decrease in the levels of this electrolyte due to hemodilution. It is important to note that, although there was a reduction in the serum concentration of this electrolyte during the 12 h of fluid therapy, the values observed in the present study remained in the reference range for horses (0.6–0.9 mmol/L) (28).

The control of urinary magnesium excretion is not yet fully understood. It was noted that the variation of the renal excretion of magnesium accompanied its serum concentration during the period of fluid therapy. Therefore, it is prudent in animals with hypomagnesemia to increase the amount of magnesium in enteral electrolyte solutions.

The small increase in SID observed at T0h, in both treatments, expresses low-intensity metabolic alkalosis, since its value in horses varies from 38 to 44 mmol/L (29, 30). This fact occurred as a consequence of the increase in serum sodium, due to water and food restriction. Despite the decrease in the SID value at T8h, in both groups, their values remained within the reference range. During the fluid therapy period (T0h–T12h) the SID values, in both treatments, were similar to the baseline values (T-12h). These results demonstrate that both treatments were able to correct the increase in SID observed at T0h. It was expected that the IsoES treatment, for containing sodium acetate, would cause an increase in the SID value, however it did not. Probably the amount of sodium acetate (4 g/L) was not enough. As the main acid-base imbalance observed in foals is metabolic acidosis with decreased SID (15), it is recommended that the enteral electrolyte solutions used to correct this imbalance in foals should contain more than 4 grams of sodium acetate per liter of solution, especially in cases of higher intensity metabolic acidosis.

The increased urinary volume and decreased urinary specific gravity of both treatment groups during fluid therapy confirm that both solutions promote volume expansion, triggering an increase in the rate of glomerular filtration. Regarding urinary specific gravity, the difference observed between treatments in the T2-6h and T10-12h period indicates that HypoES was absorbed in a greater quantity than the IsoES. These results agree with studies that evaluated the effects of enteral hypotonic solutions in adult horses (11, 14, 31).

Although there was no significant difference between the treatments (*p* > 0,05), the water volume in feces at T10–12h in the IsoES group was 900 mL/h and in the HypoES was 510 mL/h. The highest water volume in feces of the IsoES group can be attributed to the different osmolarities of the electrolytic solutions. The HypoES generated a greater osmotic gradient in the intestinal lumen, which promoted greater absorption of water by the intestine and, possibly, a higher volume expansion than that induced by the IsoES. Thus, in the IsoES group larger volume of fluid remains within the bowel loops, resulting in higher water content stools. These results agree to those observed in the urinary volume and with trials comparing the effects of hypo and isotonic enteral electrolyte solutions in adult horses (11, 12, 32). The efficacy of enteral hypotonic solutions has been observed in human patients with gastrointestinal tract infections, even in cases of secretory diarrhea (31, 33, 34). Currently, studies conducted in humans (31, 35–37) and animals (11, 35, 38) have shown that hypotonic enteral electrolyte solutions are most effective in rehydrating without causing hyponatremia. While this effect has been tested and proved in horses, cattle and calves, this is the first study that compares the effect of an IsoES and HypoES in weaned foals.

This is the first controlled study that aims to evaluate two maintenance enteral electrolytic solutions for foals under 1 year old, and for that reason, healthy animals were used in order to assess the safety of the effects of the two electrolytic solutions on hydro electrolytic balance of these animals. The authors make extensive use of enteral fluid therapy in continuous flow and the solutions evaluated in this study in the routine treatment of sick foals and both are effective in expanding blood volume and not generating iatrogenic electrolytic disturbances. Further clinical studies will be warranted to identify the specific clinical use for each solution. In addition, periodic electrolytic monitoring of foals subjected to enteral fluid therapy in continuous flow is recommended. Although we have not carried out specific analyses for the assessment of welfare, a study conducted in adult horse demonstrated lower cortisol concentration in horses with enteral fluid therapy via nasogastric tube in comparison to intravenous route (39). The foals in the present study did not show any signs of discomfort during the experiment, suggesting that the use of this technique in foals can contribute to the reduction of stress and enable better conditions of well-being for the hospitalized patient.

One of the limitations of this study was that urine collection was not performed by catheterization. However, the goal of this trial was to replicate, in controlled conditions, what the authors do in the hospital routine. Catheterization of animals for urine collection increases the stress of patients compromising their welfare and predisposes the development of urinary tract infections. For these reasons the authors chose not to use this technique in this study and the results were not compromised.

## Conclusion

The present study revealed that IsoES and HypoES electrolyte solutions tested are efficient in normalizing clinical and laboratorial parameters altered by fasting, without causing electrolyte imbalances in foals. The hypotonic enteral electrolytic solution, the HypoES, was more effective in the hydroelectrolytic reestablishment of weaned foals. These results open up new possibilities for the use of maintenance fluid therapy with hypotonic electrolyte solution in foals.

## Data Availability Statement

The datasets generated for this study are available on request to the corresponding author.

## Ethics Statement

The animal study was reviewed and approved by Ethics Committee on the Use of Animals of the Federal University of Viçosa.

## Author Contributions

LM, JR, MA, RV, and PE were responsible for the conception of the study, data analysis, and provided intellectual input on the manuscript. LM, CC, SA, PS, MS, DB, and FM were responsible for carrying out the experimental phase of the study. LM, RV, JR, PE, MA, and RT were responsible for data interpretation and writing of the manuscript.

## Conflict of Interest

The authors declare that the research was conducted in the absence of any commercial or financial relationships that could be construed as a potential conflict of interest.

## References

[1] Fielding CL Magdesian KG Edman JE. Determination of body water compartments in neonatal foals by use of indicator dilution techniques and multifrequency bioelectrical impedance analysis. Am J Vet Res. (2011) 72:1390–6. 10.2460/ajvr.72.10.1390 21962283

[2] Cohen ND. Causes of and farm management factors associated with disease and death in foals. J Am Vet Med Assoc. (1994) 204:1644–51. 8050947

[3] Galvin NP Corley KTT. Causes of disease and death from birth to 12 months of age in the thoroughbred horse in Ireland. Ir Vet J. (2010) 63:37–43. 10.1186/2046-0481-63-1-37 21851741 PMC3113843

[4] Avanza MFB Ribeiro Filho JD Lopes MAF Ignácio FS Carvalho TA Guimarães JD Enteral fluid therapy in horses - electrolyte solution associated or not with glucose, maltodextrine and magnesium sulphate: laboratory results. Ciência Rural. (2009) 39:1116–23. 10.1590/S0103-84782009005000021

[5] Ermita PAN Viana RB Ribeiro Filho JD Guimarães JD Dias DCR Monteiro BM Effects of enteral fluid therapy in continuous flow administered by nasogastric tube in buffalo calves. J Buffalo Sci. (2016) 5:60–9. 10.6000/1927-520X.2016.05.03.2

[6] Ribeiro Filho JD Avanza MFB Filho LCFB Dantas W Lima A Gomes CLN Evaluation of isotonic electrolyte solution administered by enteral via in healthy cattle or dehydrated experimentally. Vet Zootec. (2013) 20:9–16.

[7] Bregadioli G de C Pereira PFV Flaiban KKM da C Ribeiro Filho JD Lisbôa JAN Enteral fluid therapy in neonatal calves and features of commercially available electrolyte solutions in Brazil. Ciência Rural. (2017) 47:1–8. 10.1590/0103-8478cr20170140

[8] Bahl R Bhandari N Bhan MK. Reduced-osmolarity oral rehydration salts solution multicentre trial : implications for national policy. Indian J Pediatr. (1996) 63:473–6. 10.1007/BF02905721 10832467

[9] Rautanen T El-Radhi S Vesikari T. Clinical experience with a hypotonic oral rehydration solution in acute diarrhoea. Acta Paediatr. (1993) 82:52–4. 10.1111/j.1651-2227.1993.tb12516.x 8453222

[10] Ribeiro Filho JD Farias SK Donner AC Oliveira DP Guimarães JD Souza MV Horses treated with enteral electrolyte solutions with differents osmolarities. Pesqui Vet Bras. (2014) 34:179–84. 10.1590/S0100-736X2014000200014

[11] Ribeiro Filho JD de Farias SK da Fonseca LA Avanza MFB Dantas W Dias DCR Enteral electrolyte solutions with different osmolarities: clinical and laboratory assessment in equines. J Equine Vet Sci. (2015) 35:673–8. 10.1016/j.jevs.2015.06.019

[12] Ribeiro Filho JD Pessin AE Fonseca LA Dantas WMF Costa CM Ermita PAN Enteral fluid therapy in horses: effects of maintenance hypotonic electrolyte solutions containing maltodextrin, sucrose, or dextrose administered in continuous flow. J Equine Vet Sci. (2017) 50:96–101. 10.1016/j.jevs.2016.11.007

[13] Ermita PAN Ribeiro Filho JD Viana RB Silva MO Alves SR Monteiro LC Enteral fluid therapy administered in continuous flow by naso-ruminal route using three maintenance electrolyte solutions: effects on physiological biomarkers and the hemogram of bovines. Ciência Rural. (2018) 48:6–12. 10.1590/0103-8478cr20180217

[14] Dantas WMF Ribeiro Filho JD Silva GMM Ermita PAN Monteiro LC Costa CM Hypotonic enteral electrolyte solutions administered by nasoesophageal tube in continuous flow in dogs dehydrated by water restriction: Part 1. Arq Bras Med Vet Zootec. (2019) 71:404–10. 10.1590/1678-4162-10459

[15] Gomez DE Biermann NM Sanchez LC. Physicochemical approach to determine the mechanism for acid-base disorders in 793 hospitalized foals. J Vet Intern Med. (2015) 29:1395–402. 10.1111/jvim.13590 26256847 PMC4858039

[16] Constable PD Walker PG Morin DE Foreman JH. Clinical and laboratory assessment of hydration status of neonatal calves with diarrhea. J Am Vet Med Assoc. (1998) 212:991–6. 9540870

[17] Muñoz A Riber C Trigo P Castejón F. Age- and gender-related variations in hematology, clinical biochemistry, and hormones in Spanish fillies and colts. Res Vet Sci. (2012) 93:943–9. 10.1016/j.rvsc.2011.11.009 22230595

[18] Brownlow MA Hutchins DR. The concept of osmolality: its use in the evaluation of dehydration in the horse. Equine Vet J. (1982) 14:106–10. 10.1111/j.2042-3306.1982.tb02358.x 7084192

[19] Hollis AR Boston RC Corley KTT. Plasma aldosterone, vasopressin and atrial natriuretic peptide in hypovolaemia: a preliminary comparative study of neonatal and mature horses. Equine Vet J. (2008) 40:64–9. 10.2746/042516407X235795 18083662

[20] Hansen B Vigani A. Maintenance fluid therapy isotonic versus hypotonic solutions. Vet Clin North Am Small Anim Pract. (2017) 47:383–95. 10.1016/j.cvsm.2016.10.001 27908484

[21] Palmer J Practical approach to fluid therapy in neonates. 8th Int Vet Emerg Crit Care Symp. (2002) 29:665–8. 10.1136/inpract.29.3.130

[22] Harrison-Bernard LM. The renal renin-angiotensin system. Adv Physiol Educ. (2009) 33:270–4. 10.1152/advan.00049.2009 19948673

[23] Petersen KG Schluter KJ Kerp L. Regulation of serum potassium during insulin-induced hypoglycemia. Diabetes. (1982) 31:615–7. 10.2337/diab.31.7.615 6761199

[24] Ribeiro Filho JD Pessin AE Atoji K Souza MV Gomes CLN Silva AR Enteral fluid therapy: biochemical profile of horses treated with hypotonic enteral electrolyte solutions associated with energy sources. J Equine Vet Sci. (2014) 34:759–64. 10.1016/j.jevs.2014.01.004

[25] Teulon J Planelles G Sepúlveda FV Andrini O Lourdel S Paulais M. Renal chloride channels in relation to sodium chloride transport. Compr Physiol. 9, 301–42. 10.1002/cphy.c180024 30549019

[26] Hintz HF Schryver HF. Magnesium metabolism in the horse. J Anim Sci. (1972) 35:755–9. 10.2527/jas1972.354755x 5075819

[27] Alves GES Ribeiro Filho JD Oliveira HP Abreu JMG Tratamento da compactação experimental do cólon maior em eqüinos: resultados de laboratório e exames bioquímicos. Arq Bras Med Vet Zootec. (2005) 57:281–7. 10.1590/S0102-09352005000300001

[28] Toribio RE Kohn CW Rourke KM Levine AL Rosol TJ. Effects of hypercalcemia on serum concentrations of magnesium, potassium, and phosphate and urinary excretion of electrolytes in horses. Am J Vet Res. (2007) 68:543–54. 10.2460/ajvr.68.5.543 17472456

[29] Constable PD. A simplified strong ion model for acid-base equilibria: application to horse plasma. J Appl Physiol. (1997) 83:297–311. 10.1152/jappl.1997.83.1.297 9216976

[30] Navarro M Monreal L Segura D Armengou L Añor S. A comparison of traditional and quantitative analysis of acid-base and electrolyte imbalances in horses with gastrointestinal disorders. J Vet Intern Med. (2005) 19:871–7. 10.1111/j.1939-1676.2005.tb02780.x 16355683

[31] Hunt JB Thillainayagam AV Carnaby S Fairclough PD Clark ML Farthing MJG. Absorption of a hypotonic oral rehydration solution in a human model of cholera. Gut. (1994) 35:211–4. 10.1136/gut.35.2.211 8307471 PMC1374495

[32] Ribeiro Filho JD Alves GES Dantas WMF Tratamentos da compactação experimental do cólon maior de equinos com hidratação enteral, intravenosa e sene (Cassia augustifolia Vahl). Rev Ceres. (2012) 59:32–8. 10.1590/S0034-737X2012000100005

[33] Aperia A Marin L Zetterström R Günöz H Neyzi O Saner G Sökücü S. Salt and water homeostasis during oral rehydration therapy. J Pediatr. (1983) 103:364–9. 10.1016/S0022-3476(83)80404-1 6886901

[34] Farthing MJG. History and rationale of oral rehydration and recent developments in formulating an optimal solution. Drugs. (1988) 36:80–90. 10.2165/00003495-198800364-00011 3069448

[35] Nishinaka D Kishino F Matsuura A Water and electrolyte absorption from hypotonic oral rehydration solution in rat small intestine and colon. Pediatr Int. (2004) 46:315–21. 10.1111/j.1442-200x.2004.01887.x 15151549

[36] Rautanen T Kurki S Vesikari T. Randomised double blind study of hypotonic oral rehydration solution in diarrhoea. Arch Dis Child. (1997) 76:272–4. 9135272 10.1136/adc.76.3.272 PMC1717093

[37] Hunt JB Thillainayagam A V Salim AFM Carnaby S Elliott EJ Farthing MJG Water and solute absorption from a new hypotonic oral rehydration solution: evaluation in human and animal perfusion models. Gut. (1992) 33:1652–9. 10.1136/gut.33.12.1652 1487167 PMC1379577

[38] Sosa León LA Davie AJ Hodgson DR Rose RJ. The effects of tonicity, glucose concentration and temperature of an oral rehydration solution on its absorption and elimination. Equine Vet J. (1995) 27:140–6. 10.1111/j.2042-3306.1995.tb05020.x 8933097

[39] Gomes CLN Ribeiro Filho JD Farias SK Donner AC Effects of PEG 3350 or an enteral solution associated or not with Ringer lactate, and of NaCl 0.9% on the glucose, lactate, cortisol and insulin of healthy equines. Arq Bras Med Vet Zootec. (2014) 66:1039–45. 10.1590/1678-5643

